# Similarity of DMD gene deletion and duplication in the Chinese patients compared to global populations

**DOI:** 10.1186/1744-9081-4-20

**Published:** 2008-04-29

**Authors:** Xiaozhu Wang, Zheng Wang, Ming Yan, Shangzhi Huang, Tian-Jian Chen, Nanbert Zhong

**Affiliations:** 1 Peking University Center of Medical Genetics, Peking University, Beijing, China; 2 Department of Medical Genetics, Peking University Health Science Center, Peking University, Beijing, China; 3 Department of Medical Genetics, Peking Union Medical College/Institute of Medical Sciences, Chinese Academy of Medical Sciences, Beijing, China; 4 Department of Human Genetics, New York State Institute for Basic Research in Development Disabilities, Staten Island, New York, USA; 5 Department of Medical Genetics, University of South Alabama, Mobile, Alabama, USA

## Abstract

**Background:**

DNA deletion and duplication were determined as the major mutation underlying Duchenne muscular dystrophy (DMD) and Becker muscular dystrophy (BMD).

**Method:**

Applying multiplex ligation-dependent probe amplification (MLPA), we have analyzed 179 unrelated DMD/BMD subjects from northern China.

**Results:**

Seventy-three percent of the subjects were found having a deletion (66.25%) or duplication (6.25%). Exons 51–52 were detected as the most common fragment deleted in single-exon deletion, and the region of exons 45–50 was the most common exons deleted in multi-exon deletions. About 90% of DMD/BMD cases carry a small size deletion that involves 10 exons or less, 26.67% of which carry a single-exon deletion. Most of the smaller deletions resulted in an out-of-frame mutation. The most common exons deleted were determined to be between exon 48 and exon 52, with exon 50 was the model allele. Verifying single-exon deletion, one sample with a deletion of exon 53 that was initially observed from MLPA showed that there was a single base deletion that abolished the ligation site in MLPA. Confirmation of single-exon deletion is recommended to exclude single base deletion or mutation at the MLPA ligation site.

**Conclusion:**

The frequency of deletion and duplication in northern China is similar to global ethnic populations.

## Background

Duchenne muscular dystrophy (DMD) and its less severe allelic form Becker muscular dystrophy (BMD) are common X-linked recessive neuromuscular degeneration diseases [1,2]. The incidence of DMD is 1/3500 and BMD is 1/18,000 in male living births, respectively [3,4]. They both are caused by genetic defects of the dystrophin gene, which is located at Xq21.2, spanning 2.4 Mb [5,6]. The dystrophin gene consists of 79 exons and encodes a 14.6 kb mRNA, which is expressed in skeleton, muscles and brain [7]. Deletions, duplications and point mutations have been reported in most exons of this gene. There were two deletion hot-spot regions, one is located at the 5' exons 2–20 and the other, located between exons 44–53 [8,9]. Mutations affecting open-reading frame (ORF), due to a frameshift, may result in an aberrantly spliced mRNA and generate a truncated, nonfunctional dystrophin protein that usually gives rise to the DMD/BMD phenotypes. However, mutations not affecting the ORF may produce a semi-functional dystrophin protein and usually correlate with mild phenotypes [10]. Levels of dystrophin protein in DMD are very low (<3% of normal) or absent, however, BMD patients have 10–40% of the normal amount of dystrophin with an abnormal molecular weight [11,12]. About 65% of cases are caused by deletion [8,9,13], approximately 5% by duplication [9,14] and the remaining is point mutation of the dystrophin gene [15]. Due to no effective treatment available for DMD/BMD, presently an accurate genetic diagnosis may offer prenatal diagnosis for the familial DMD/BMD although about one-third of mutations are *de novo *[16]. Although the frequency of complex rearrangement was reported as high as 4% [17], usually complex rearrangement is considered as a rare condition [18-20] that includes double deletion [17] and non-contiguous duplications [21-23].

In this study, we have analyzed a subset of DMD/BMD patients from Northern China.

## Methods

### Subjects

A total number of 179 unrelated patients in northern China were studied. There were 160 affected male probands and 19 females who had an affected son clinically diagnosed as DMD/BMD. This study was approved by the Institutional Ethic Committee and informed consent was obtained from all participants.

### Multiplex ligation-dependent probe amplification (MLPA)

MLPA is a reliable technology for quantitative analysis of the copy number in clinical diagnosis of genetic diseases [24]. It has the capability of detecting either large or small genomic deletions or duplications [20,25]. An MLPA kit with probes of P034 and P035 for detecting deletion and/or duplication of the DMD gene was purchased commercially from MRC Holland (Amsterdam, Netherlands). Procedures were following the manufacturer's instruction. Ligation and amplification were carried out with an ABI 9800 Thermal Cycler. The PCR conditions were 35 cycles at 95°C for 30s, 60°C for 30s, and 72°C for 60s; followed by a final incubation at 72°C for 20 min.

**Figure 1:**
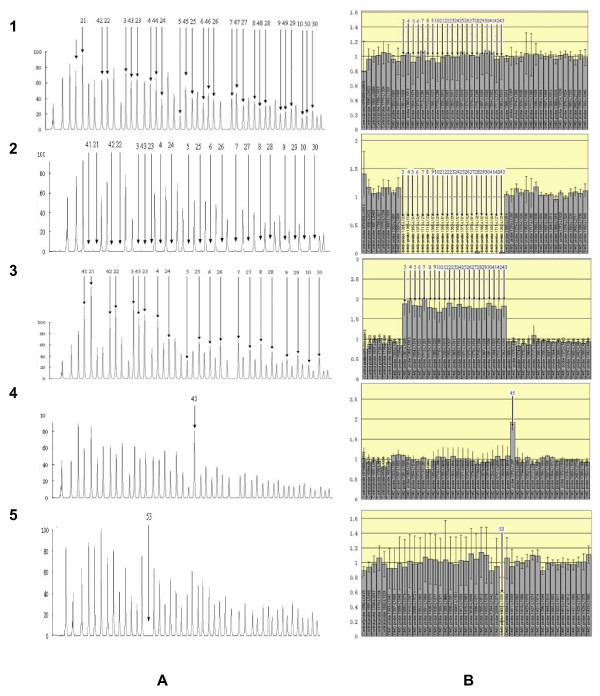
MLPA results were selected from a normal control (1), a multi-exon deletion (2), a multi-exon duplication (3), a single-exon duplication (4), and a single-exon deletion (5). Raw data are shown on the left panels (A). Each peak represents one exon of the dystrophin gene. Arrow(s) indicate missing signals representing deleted exon(s). The corresponding relative peak area (RPA) ratios, after statistical analysis, are shown on the right panels (B), in which each bar represents the RPA ratio compared to the mean of two male control samples.

The PCR products were separated by capillary electrophoresis in an ABI 310 Genetic Analyzer with Genescan 3.7 (Applied Biosystems, Foster City, California) software. The raw data was analyzed by Genescan Analysis 3.7 software. The peaks obtained after the analysis of the row data (Fig. 1A) could be distinguished and assigned to specific exons on the basis of their different lengths representing the variability of their stuffer sequences. Peak area of raw data can be also exported into a Microsoft excel spreadsheet program (Fig. 1B), which normalizes each peak to that of a known normal control (Fig. 1, 1). Peaks derived from DMD/BMD patients (Fig. 1, 2–5) that vary more than 20% from the normal should be flagged for review. If a deletion of a single exon was observed, conventional PCR and DNA sequencing analysis of the specific region embedding the deleted exon was performed to verify the deletion [20,21].

## Results

### Detection of DMD gene deletion with MLPA

Among 160 male probands, 106 were found to have deletions, accounting for 66.25% of male patients analyzed. Among 19 females who had an affected boy but without previous genetic confirmation, 14 with exonic deletion were detected, which brought the deletion rate to 67.04% (120/179). Duplication mutation was identified from 10 (16.95%) of the remaining 59 people (54 male patients and 5 female carriers) who had no deletion identified. Totally, 130 of 179 (72.63%) were found to have mutations (Table 1).

**Table 1:** Mutation Detected in Dystrophin Gene

**Size of deletion or duplication**	**Independent cases (N = 120)**	**Distribution (%)**	**Exon deleted or duplicated**	**Fragment deleted or duplicated Δ**	**IF/OF OF**	**Minimal deletion/duplication (bp)**
1 exon (n = 32)	1	0.83	DelEX07	c.531-?*_649+?*del	OF	118
	1	0.83	DelEX17	c.1993-?_2168+?del	OF	175
	2	1.67	DelEX18	c.2169-?_2292+?del	OF	123
	1	0.83	DelEX19	c.2293-?_2380+?del	OF	?
	1	0.83	DelEX35	c.4846-?_5025+?del	IF	179
	2	1.67	DelEX43	c.6118-?_6290+?del	OF	172
	1	0.83	DelEX44	c.6291-?_6438+?del	OF	147
	5	4.17	DelEX45	c.6439-?_6614+?del	OF	175
	2	1.67	DelEX50	c.7201-?_7309+?del	OF	108
	6	5	DelEX51	c.7310-?_7542+?del	OF	232
	6	5	DelEX52	c.7543-?_7660+?del	OF	117
	2	1.67	DelEX53*	c.7661-?_7872+?del	OF	211
	2	1.67	DelEX54	c.7873-?_8027+?del	OF	154
2 exons (n = 10)	1	0.83	DelEX10-11	c.961-?_1331+?del	OF	1020
	1	0.83	DelEX42-43	c.5923-?_6290+?del	OF	22747
	2	1.67	DelEX46-47	c.6615-?_6912+?del	OF	2631
	4	3.33	DelEX48-49	c.6913-?_7200+?del	IF	38655
	2	1.67	DelEX49-50	c.7099-?_7309+?del	OF	104
3 exons (n = 17)	1	0.83	DelEX03-05	c.94-?_357+?del	IF	26525
	2	0.83	DelEX46-48	c.6615-?_7098+?del	OF	57036
	6	5	DelEX48-50	c.6913-?_7309+?del	OF	55398
	2	1.67	DelEX50-52	c.7201-?_7660+?del	OF	90452
	3	2.5	DelEX51-53	c.7310-?_7872+?del	OF	94817
	1	0.83	DelEX52-54	c.7543-?_8027+?del	OF	71758
	2	1.67	DelEX53-55	c.7661-?_8217+?del	OF	51913
4 exons (n = 9)	1	0.83	DelEX10-13	c.961-?_1602+?del	IF	49395
	1	0.83	DelEX22-25	c.2804-?_3432+?del	OF	8870
	3	2.5	DelEX45-48	c.6439-?_7098+?del	IF	93323
	1	0.83	DelEX46-49	c.6615-?_7200+?del	OF	95509
	2	1.67	DelEX49-52	c.7099-?_7660+?del	OF	107011
	1	0.83	DelEX50-53	c.7201-?_7872+?del	IF	140708
5 exons (n = 12)	2	1.67	DelEX03-07	c.94-?_649+?del	OF	40327
	1	0.83	DelEX13-17	c.1483-?_2168+?del	OF	50717
	1	0.83	DelEX17-21	c.1993-?_2803+?del	OF	60415
	3	2.5	DelEX46-50	c.6615-?_7309+?del	OF	102252
	3	2.5	DelEX48-52	c.6913-?_7660+?del	OF	145742
	1	0.83	DelEX50-54	c.7201-?_8027+?del	OF	162093
	1	0.83	DelEX51-55	c.7310-?_8217+?del	OF	146519
6 exons (n = 13)	7	5.83	DelEX45-50	c.6439-?_7309+?del	OF	148539
	1	0.83	DelEX46-51	c.6615-?_7542+?del	OF	158627
	1	0.83	DelEX47-52	c.6763-?_7660+?del	OF	200114
	4	3.33	DelEX49-54	c.7099-?_8027+?del	OF	178832
7 exons (n = 2)	2	1.67	DelEX46-52	c.6615-?_7660+?del	OF	202596
8 exons (n = 5)	4	3.33	DelEX45-52	c.6439-?_7660+?del	OF	238883
	1	0.83	DelEX46-53	c.6615-?_7872+?del	OF	252852
10 exons (n = 6)	3	2.5	DelEX45-54	c.6439-?_8027+?del	OF	310524
	3	2.5	DelEX46-55	c.6615-?_8217+?del	OF	304554
11 exons (n = 3)	3	2.5	DelEX45-55	c.6439-?_8217+?del	IF	340841
12 exons (n = 1)	1	0.83	DelEX31-42	c.4234-?_6117+?del	IF	80099
16 exons (n = 1)	1	0.83	DelEX8-23	c.650-?_3162+?del	OF	230795
20 exons (n = 1)	1	0.83	DelEX11-30	c.1150-?_4233+?del	IF	232561
27 exons (n = 1)	1	0.83	DelEX3-29	c.832-?_3162+?del	IF	411579
30 exons (n = 1)	1	0.83	DelEX10-39	c.961-?_5586+?del	IF	299209
35 exons (n = 1)	1	0.83	DelEX10-44	c.961-?_6438+?del	IF	428236
37 exons (n = 1)	1	0.83	DelEx8-44	c.650-?_6438+?del	OF	482377
39 exons (n = 1)	1	0.83	DelEX3-41	c.94-?_5922+?del	IF	507720
41 exons (n = 1)	1	0.83	DelEX3-43	c.94-?_6290+?del	OF	562291
42 exons (n = 2)	2	1.67	DelEX3-44	c.94-?_6438+?del	IF	632904
Dup1 exons	1	0.83	DupEX45	c.6439?_6614+?dup	OF	175
Dup3 exons	1	0.83	DupEX48-50	c.6913?_7309+?dup	OF	55398
Dup5 exons	1	0.83	DupEX3-7	c.94-?_649+?dup	OF	40327
Dup11 exons	1	0.83	DupEX64-74	?	OF	53678
Dup14 exons	1	0.83	DupEX3-16	c.94-?_1992+?dup	IF	284118
Dup21 exons	1	0.83	DupEX54-74	c.7873-?_10553+?dup	OF	488701
Dup27 exons	1	0.83	DupEX8-34	c.650-?_4845+?dup	OF	318783
Dup29 exons	1	0.83	DupEX13-41	c.1483-?_5922+?dup	IF	253776
Dup37 exons	1	0.83	DupEX8-44	c.650-?_6438+?dup	OF	482377
Dup41 exons	1	0.83	DupEX3-43	c.94-?_6290+?dup	OF	562291

***Δ: ***data were obtained through online search from [26].

?: the actual breakpoint is unknown.

**Figure 2:**
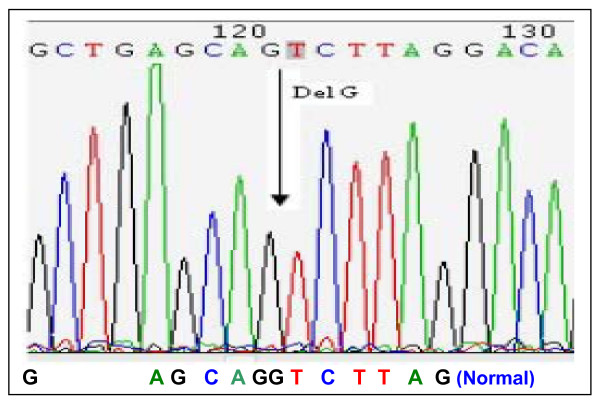
Sequencing analyses to validate single-exon deletion. One sample detected as a single-exon deletion of exon 53 was found that the deletion was resulted from a single base pair deletion, rather than an entire exon deletion. This single base pair deletion abolished the ligation of the probe.

*: DNA sequencing analysis determined that this single exon deletion is due to a single base pair deletion (Fig. 2).

IF/OF: in frame or out-of-frame.

### Detection of single-exon deletion may result from 1-bp deletion

When a single-exon deletion was observed, DNA sequencing would be applied for verification. Among 32 DNA samples with single-exon deletion, DNA sequencing observed a 1-bp deletion (NM_004006.1, c.8027delG) in one case that had a deletion of exon 53 detected by initial MLPA. This 1-bp deletion (Fig. 2) was located precisely at the ligation site and destroyed the ligation that resulted in failure of amplification of the probes in MLPA.

### Deletion of small fragment is predominant in DMD gene

Analyses of 106 males and 14 females (Table 1) showed that 26.67% (29 males and 3 females) of deletions involved a single-exon; 14.17% (15 males and 2 females) exhibited 3-exon deletion; 10.83% (12 males and 1 female) had 5- or 6-exon deletion, 8.33% (10 males) had 2-exon deletion, 7.50% (5 males and 4 females) showed 4-exon deletion, 5.0% (6 males) had 10-exon deletion, 4.17% (5 males) had 8-exon deletion, and 1.67% (2 males) had 7-exon deletion. Our results showed that deletion of six exons and smaller accounts for 77.5% (93/120). To combine all deletions smaller than 10 exons, this group deletion may account for 88.33% (106/120) of patients and carriers.

### Duplication detected in this study involved a large fragment

Ten patients with duplication were identified. Most of the duplications involved large fragments, among which six spun 11 to 41 exons. Eight duplications involved previously identified 3' region. A large 21-exon duplication was beyond the 3' hot-spot region that has reached exon 74. Only three duplications in this study were found to involve small fragment.

### Exons 48–52 were the most common region deleted

**Figure 3:**
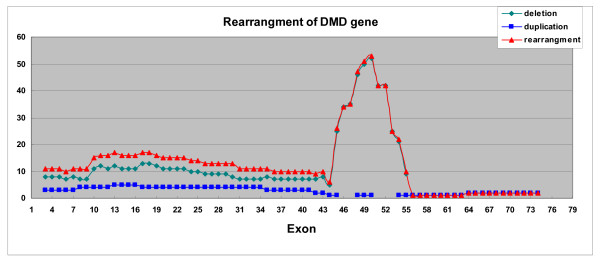
Frequency of DMD gene mutations showed that deletion of exon 50 is the most common in the DMD gene deletions, which brought the exon 50 as the model allele in the DMD gene deletion. There is no specific peak showing a model duplicated exon.

Analysis of the single-exon deletion among the 32 (29 males, three females) patients showed that both exons 51 and 52 were the most common exons deleted, accounting for 18.75% (6/32) respectively, followed by exon 45 (15.63%). Single-exon deletion was not found in exons 1–6, 8–16, 20–34, 36–42, 46–49, and 55–79. Among the 88 cases with multiple exons (multi-exon), deletion of six exons in 45–50 were the most common (7.95%), followed by deletion of three exons in 48–50 (6.82%), and then two exons in 48–49 and eight exons in 45–52. When the frequency of deletion for individual exon of dystrophin gene was considered, the most common deleted was exon 50, followed by exon 49 (6.54%), then exon 48 (6.01%), and exons 51 (5.10%). Deletion of exons from 45 to 54 accounts for 50.16%. No deletion was found in either 5' end exons 1–2 or 3' end exons 56–79 (Fig. 3).

### Out-of-frame mutation is predominant in DMD/BMD

It has been considered that clinical severity is correlated to in frame or out-of-frame deletion of the dystrophine gene [26]. An interesting result we noticed from this study was that for smaller deletions, they usually resulted in out-of-frame mutations. Among 106 small deletion that involved ten or less exons, there were only 11 cases that carried in-frame but 89.62% had out-of-frame. However, among 14 larger deletions, there were 11 (78.57%) cases had in-frame mutations. This data suggested that out-of-frame mutations in smaller deletion cases account for the majority of DMD mutations, which likely resulted in a severe phenotype. Among five larger duplications, there was only one that was an in-frame mutation while the others were out-of-frame mutations, and there was no in-frame mutation in two small duplications. Overall, 81.53% of duplications were out-of-frame. Correlating each DNA sample with its clinical phenotypes would be idea for drawing a clearer picture. A regret of this study, unfortunately, was that the clinical information had been incomplete which made us unable to perform an analysis on genotype-phenotype correlation. We encourage to perform a genotype-phenotype study with a larger cohort in order to draw a better clue.

## Discussion

Several studies have shown that MLPA were useful in detecting mutation quantitatively, not only for deletions but also duplications and female carriers in the DMD gene [20,25,27-29]. We applied MLPA in a subset of DMD/BMD patients in northern China. Our results indicated that 66.25% (106/160) of our patients carry deletion and 6.25% (10/160) patients had a duplication, which brought the sensitivity of detecting DNA rearrangement up to 72.5%. In addition, MLPA may present higher sensitivity. In this study, three patients who had negative results in their previous DNA testing by multiplex PCR (data not shown) were identified. Furthermore, an advantage of employing MLPA in clinical genetic testing is that it is capable of identifying female carriers, as we have demonstrated among 14 out of 19 females who came to our prenatal clinics. Certainly, MLPA should be considered as the initial test for the clinically suspected DMD/BMD patients as well as for women who have a DMD/BMD family history, as suggested previously [29], to provide both confirmation of genetic defects on DMD gene and better genetic counseling.

**Figure 4:**
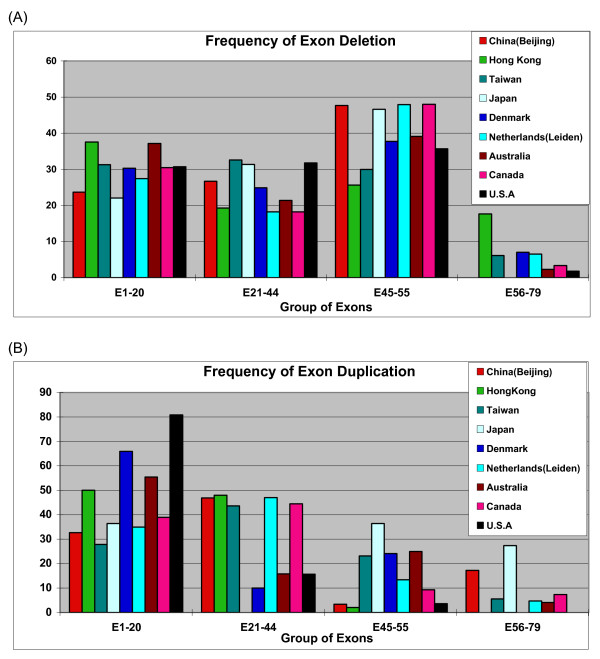
Comparison of exon deletion (A) and duplication (B) among global populations. All these data showed that exons 45–55 are the most frequent deletion region of DMD gene. Deletion and duplication were not common in region of exons 56–79, although the data may derive from variant methodology (MLPA for Hong Kong, Taiwan, Denmark, Australia, and the current study; quantitative multiplex PCR for Canada; and multiplex PCR and Southern blotting for Japan and USA).

Exons 2–20 and exons 44–53 had been previously reported as the two-hotspot regions [8,9]. In this study, we have analyzed the distribution of individual exons regarding their frequency of deletion or duplication (Fig. 4). Our data indicates that the deletion occurs at exons 45 to 55 accounting for 47.67%, followed by exons 21–44 (26.67%) and exons 1–20 (23.66%). If both deletions and duplications are counted together, the most frequent mutant region occurs within exons 45–55 (41.06%) in the DMD gene, followed by exons 21–44 (30.43%), exons 1–20 (25.32%) and exons 56–79 (3.19%). We found the most common region in our study is the exons 45–55, and the least common region is the exons 56–79. These data are in agreement with other global populations [26]. We have noticed that the frequency of duplication is more than 20% and it appears there is no model peak of deletion in Taiwan (Fig. 4) [21,22,27,30-32]. As well, the deletion rate in Hong Kong [31] showed difference from the northern Chinese. We hypothesize that the frequency of deletion is different in variant regions of China, and we propose that further investigation with molecular screening of deletion (and duplication) should be pursued among newborns in China. The pattern of duplication did not show significant difference among the global variant populations (Fig. 4).

The structure of introns of dystrophin gene determines the breakpoint that is responsible for the deletion [33]. There were many repeated sequences in introns of dystrophin gene which resulted in many breakpoints. For example, in intron 44 there were 13 breakpoints resulted in the hot-spot deletion region [34]. The repeated sequences included direct repeated sequence, inverted repeat sequence and homology sequence. It was reported that 22 repeated sequences had been found in intron 51 and intron 52, which was a possible reason for the high frequency of single-exon deletion at exon 51 [35]. We believe this might be the same case for the most common deletion at exon 50.

Recently, White et al [25] reported that as high as 87% of duplications can be detected among three subsets of selected DMD/BMD cases, among which no deletion or point mutation had been found. In this study, duplication was only detected in 18.51% of the cases where no deletion could be detected. This lower rate possibly resulted from either our small size of samples or a high rate of point mutation, which should be further investigated. To combine the rates of duplication with deletion, we estimate that a point mutation might account for 20–28% of our cases. In addition, the duplications in our cases mainly involved a larger fragment; however, among 118 cases of the reported duplications [25], there were only 12 cases where the duplication was more than 20 exons.

In summary, we have applied MLPA to detect DNA rearrangements of deletions and duplications among DMD/BMD male patients and female potential carriers. We have determined that deletions and duplications were 72.5% of our samples. We observed that the majority of deletions was small in size that the number of deleted exons was less than 10 but duplications usually involved larger sizes. Finally, we determined that the most common region of DMD gene rearrangement is between exons 45 and 55 and the 5' hotspot region was not a hotspot region in our study.

## Conclusion

MLPA is an effective and cost effective technique to detect a large number of samples. In our study, we found the frequency of deletion/duplication of DMD in the northern China is similar to other population. We recommend that MLPA should be applied as the first line for clinical molecular testing for DMD/BMD.

## List of abbreviations

DMD: Duchenne muscular dystrophy; BMD: Becker muscular dystrophy; MLPA: multiplex ligation-dependent probes amplification; PCR: ploymerase chain reaction.

## Competing interests

The authors declare that they have no competing interests.

## Authors' contributions

XZW conducted experiments, analyzed and interpreted results, and drafted the manuscript. ZW assisted in data analysis and manuscript drafting. SZH participated in DNA sample collection. MY assisted in operating fragment analysis with sequencer ABI310. TJC directed raw data analysis. NZ conceived of the project, designed, supervised, and coordinated the entire studies, and is responsible for the final edition of the manuscript and approval of the submission. All authors read and approved the final manuscript.
